# The non-linear risk of mortality by income level in a healthy population: US National Health and Nutrition Examination Survey mortality follow-up cohort, 1988–2001

**DOI:** 10.1186/1471-2458-8-383

**Published:** 2008-11-10

**Authors:** David H Rehkopf, Lisa F Berkman, Brent Coull, Nancy Krieger

**Affiliations:** 1 Department of Epidemiology and Biostatistics, University of California, San Francisco, 185 Berry Street, Lobby 5, Suite 5700, Campus Box 0560, San Francisco CA 94118, USA; 2 Department of Society, Human Development and Health, Harvard School of Public Health, 677 Huntington Ave, Boston MA 02115, USA; 3 Department of Biostatistics, Harvard School of Public Health, 677 Huntington Ave, Boston MA 02115, USA

## Abstract

**Background:**

An examination of where in the income distribution income is most strongly associated with risk of mortality will provide guidance for identifying the most critical pathways underlying the connections between income and mortality, and may help to inform public health interventions to reduce socioeconomic disparities. Prior studies have suggested stronger associations at the lower end of the income distribution, but these studies did not have detailed categories of income, were unable to exclude individuals whose declining health may affect their income and did not use methods to determine exact threshold points of non-linearity. The purpose of this study is to describe the non-linear risks of all-cause and cause-specific mortality across the income distribution.

**Methods:**

We examined potential non-linear risk of mortality by family income level in a population that had not retired early, changed jobs, or changed to part-time work due to health reasons, in order to minimize the effects of illness on income. We used data from the US National Health and Nutrition Examination Survey (1988–1994), among individuals age 18–64 at baseline, with mortality follow-up to the year 2001 (ages 25–77 at the end of follow-up, 106 037 person-years of time at risk). Differential risk of mortality was examined using proportional hazard models with penalized regression splines in order to allow for non-linear associations between mortality risk and income, controlling for age, race/ethnicity, marital status, level of educational attainment and occupational category.

**Results:**

We observed significant non-linear risks of all-cause mortality, as well as for certain specific causes of death at different levels of income. Typically, risk of mortality decreased with increasing income levels only among persons whose family income was below the median; above this level, there was little decreasing risk of mortality with higher levels of income. There was also some variation in mortality risk at different levels of income by cause and gender.

**Conclusion:**

The majority of the income associated mortality risk in individuals between the ages of 18–77 in the United States is among the population whose family income is below the median (equal to $20,190 in 1991, 3.2 times the poverty level). Efforts to decrease socioeconomic disparities may have the greatest impact if focused on this population.

## Background

Despite longstanding knowledge of an inverse association between income and mortality in the United States [1,2] and calls to reduce socioeconomic gradients in health [3], few studies have examined whether the higher risk of mortality at lower incomes is uniform across the income distribution or whether threshold points exist in this association. Several studies from the UK and Scandinavia suggest that a threshold point does not exist in the association between social class and mortality [4-6], and this has been used to inform policy for reducing health disparities worldwide [7]. A linear risk of mortality across the income distribution implies that there are etiologic pathways from income to mortality for all individuals, regardless of income level. Conversely, studies from the US and New Zealand have found primarily non-linear associations between income and mortality [8-10]. If the association is non-linear and the income-mortality gradient exists mainly at lower income levels, research to understand income differences in health should be among this population. In addition to guiding etiologic research of income-mortality associations, the shape of association has implications for efforts to reduce income-based disparities: either providing evidence in support of targeting the full population (if a linear association) or evidence for support of policies focused on the population where such associations are found to exist.

Within the US, only two studies have explicitly examined the shape of the relationship between income and mortality [9,10] and both found suggestive evidence of non-linear associations, with a higher level of income more strongly associated with increased mortality at the lower end of the income distribution. These studies were limited, however, by: (1) a lack of access to finely-measured income data (thus unable to determine if threshold points exist in the risk of mortality); (2) an inability to exclude persons whose declining health status may have reduced their income level [11], and (3) methods that did not allow for the testing of statistically significant non-linear associations and threshold points in change in mortality risk.

The aim of this paper is to describe the shape of the income and all-cause and cause-specific mortality associations among US adults age 18 to 64 at baseline (who were age 25–77 by the end of follow-up). We examined the association of income and mortality, restricting our analysis only to those individuals who were free from health conditions that caused them to change jobs, change to part time work, or retire early due to health reasons. By using data with a large number of income categories and by modelling the association without using a pre-specified functional form or pre-specified inflection points we are able to more accurately estimate the shape of the income and cause-specific mortality associations. We also compare the fit of models with baseline covariates and either a linear income term, a log-income term, or a smoothed spline income term in order to determine which income-mortality model provides the best fit to the data.

## Methods

The US Third National Health and Nutrition Examination Survey (NHANES III), 1988–1994, was designed to be representative of the non-institutionalized population of the U.S. when analyzed using weights to account for over-sampling and non-response [12]. Our analysis used data from the National Death Index (NDI), for deaths through December 31, 2001 linked to NHANES III data, for a length of follow up of up to 13 years. The NDI is a well documented and validated method of matching deaths in the United States to population datasets [13,14]. The definition of cause of death has been validated to have a discrepancy rate of approximately 5% [15]. Only NHANES III participants at least 18 years of age at the time of interview were eligible for follow-up, and we limited our analyses to individuals that were under the age of 65 at baseline (n = 14,798) (individuals missing at the time of follow up (0.3%) were excluded). We excluded the following individuals to minimize causation from health to income: individuals who self-reported that they "changed permanently to an easier job," "changed temporarily to an easier job," "cut down to part-time work only," "have to stop working for a few months," or "retire because of a disability," for the reason of "a disability or health problems" (10%) [16]. We also excluded individuals who died after one year or less of follow up (0.3%), as illness in the year prior to death may have reduced family income. Individuals who were missing income (7%), education (0.05%) or occupation (1%) were also excluded. The sum of these exclusions resulted in a final analytic sample of 11,733 individuals (106 037 person-years) with 349 deaths (table 1).

**Table 1:** Demographic characteristics of full and restricted analytic sample, ages 18–64, NHANES, 1988–2001

	**Full sample (n = 14,798)**		**Restricted analytic sample (n = 11,733)**	
	***percent***	***std error***	***percent***	***std error***
***Age***				
18–24	17	0.6	18	0.6
26–44	53	0.9	55	1.0
45–64	30	0.8	28	0.9
***Gender***				
Men	49	0.4	49	0.5
Women	51	0.4	51	0.5
***Race/ethnicity***				
White	74	1	75	1
Black	12	0.6	11	0.6
Mexican-American	6	0.5	6	0.5
Other	8	0.9	8	1
***Marital status***				
married/living as	64	0.9	64	0.9
not married/not living as	36	0.9	36	0.9
***Education***				
< high school	23	1	22	1
high school	35	0.8	34	0.8
> high school	42	1	44	1
***Occupation***				
white collar and professional	24	0.9	25	0.9
white collar, semi-routine	23	0.7	23	0.7
blue collar, high skill	12	0.5	12	0.5
blue collar, semi-routine	39	0.8	38	0.9
never worked	3	0.3	3	0.3
***Income (equivalized for family size)***				
< 50% median	22	1	21	1
50–100% median	31	1	30	1
> median	47	2	49	2
% below poverty	11	0.7	11	0.8
% poverty to 184% poverty	16	0.8	15	0.8
185% poverty to median income*	25	1	25	1
> median*	47	2	49	2
***Work and health***				
not currently in labor force	25	0.7	21	0.7
employment affected by health	10	0.4	0	-
death with <1 year follow-up	0.3	0.07	0	-

*median income is equal to 322% of the poverty line

We examined all-cause mortality and three cause-specific categories of adult mortality as defined by the following ICD-10 classifications: 1) heart disease (I00-I09, I11, I13, I20-I51), 2) cancer (C00-C97) and 3) injury (both intentional and unintentional) (V01-Y34, Y85-Y86, Y87.0, Y87.1, Y87.2, Y89.9).

Total combined pre-tax family income for the 12 months prior to the survey included wages, salaries, income from self-employment, veteran's benefits, interest dividends, rental income and public assistance. Family income data were available in 28 income categories (none, less than $1000, $1000 to $20,000 in $1000 dollar increments, $20,000 to $50,000 in $5000 dollar increments, and greater than $50,000). Income from each half of the survey (1988–1991, 1991–1994) was adjusted to 1991 dollars using the Urban Consumer Price Index. For all analyses we used the midpoint of each income category and calculated the mid-point of the upper category of income ($66,800) by assuming a Pareto distribution of family income per standard methodology [17]. Income (as well as reference points of median income and poverty line) is made equivalent for economies of scale by dividing family income by the square root of family size. In this study we conceptualize income as a measure of the level of earnings of a household at a particular point in time, a value that has been empirically shown to have a strong association with lifetime earnings[18]

Additional covariates included: (a) education (0–17 or more years), (b) race/ethnicity (using the available categories of white non-Hispanic, Hispanic, black, other), (c) age (in years), and (d) occupation, referring to the longest held occupation, divided into 5 categories: (1): white collar and professional (e.g. executive, management, professional, supervisors); (2): white collar, semi-routine (e.g. technicians, sales workers, secretaries); (3): blue collar, high skill (e.g. mechanics, construction trades, military); (4): blue collar, semi-routine (e.g. personal services, waiters and waitresses, food preparation, farm workers, motor vehicle operators); and (5): never worked. Detailed NHANES III occupational categories were used to create this variable [see Additional file 1]. Labor force participation was defined as working at a job or business or having a job or business within the last two weeks, not including work around the house.

In order to be consistent with prior work on the shape of the association of income and mortality [6,8,9] we modelled mortality for women and men separately. The three models that we present include the following covariates: model 1) income, age, race/ethnicity, marital status, model 2) income, age, race/ethnicity, marital status, education, occupation, model 3) income, age, race/ethnicity, marital status, education, occupation, labor force participation. The estimated income coefficient from model 1 is not conditional on other measures of socioeconomic position, and thus may not adequately control for confounding of the income-mortality association. The estimated income coefficient from model 2 is conditional on other measures of socioeconomic position (i.e. education and occupation), and is the primary model for inference and for which associations are shown in the figures. Model 3 additionally controls for current labor force participation, as this may confound the association of income and mortality, but may also be on the causal pathway between income and mortality and thus should not be included as a control variable [8].

The income and cause-specific mortality associations were modelled with penalized splines (with a cubic basis) in proportional hazard survival models in order to allow for possible non-linear dependence of mortality hazard on income (as well as for education when included as a covariate)[19,20]. We use age as the time-scale of the baseline hazard rather than time-on-study as this allows for less biased model estimates with respect to age, and allows for non-linear associations between age and mortality [21,22]. Penalized splines model the association by fitting a large number of regression splines joined at evenly spaced knots, with a penalty on coefficients of adjacent knots [23]. The starting point for the model fitting procedure is to fit a saturated model of a large number of splines, and then to penalize the fit based on a smoothing parameter (theta) to adjust the penalty term to avoid over fitting the data. Prior work has shown that by using this method smoothed estimates of survival are insensitive to the number and location of knots [23]. Theta can vary between 0 and 1, where 0 is close to an exact fit to the data, and 1 is a straight line. The initial values of theta were chosen by generalized cross validation – an automated model fitting procedure where the optimal value of theta is determined by repeatedly fitting the model on random subsets of the data and then testing how well the model predicts the data that was not used to create the model [20]. Based on subject matter knowledge, we then increased the thetas to be slightly closer to 1 (a more conservative model) to avoid models that overfit the data, although fit was very similar in both sets of models (model fit from the theta chosen by generalized cross validation results available from the authors). All models we present are thus more conservative models for identifying non-linearity then those selected by generalized cross-validation.

We first present the unadjusted incidence rates of all-cause mortality by gender and income (table 2). We then present the Chi-square statistics and associated two-sided p-values from adjusted spline models in order to allow for assessment of the degree of difference in spline slopes for income parameters, values that may be interpreted as tests of non-linearity (table 3) [20]. We also present likelihood ratio tests comparing fitted models with a spline term to: 1) models controlling for baseline covariates, 2) models controlling for baseline covariates and a linear income term, and 3) models controlling for baseline covariates and log transformation of income (table 4). A likelihood ratio test comparing each fitted model to a null model is used to determine overall model fit. The null hypothesis for this comparison is no difference in model fit, therefore p-values of less than 0.05 reject the null hypothesis and indicate that the fitted model is a better description of the data. Due to the different degrees of freedom in each of the models, it is most appropriate to compare p-values across models to evaluate relative model fit. All analyses were conducted using R version 2.5.1[24] The proportional hazard model was implemented using the function coxph(), and the penalized spline fit was implemented using the pspline() function[25] Plotted standard errors were calculated by a grouped jackknife estimate implemented using the cluster() function, and all models employed weighting to account for the complex sampling design. Survey adjusted population frequencies and histograms were calculated using the survey package [26,27]. This research was approved by the Harvard School of Public Health's Committee on Human Subjects.

**Table 2:** Unadjusted all-cause mortality rates by category of family equivalized income, healthy restricted population, ages 18–64, NHANES, 1988–2001

	**Women**				**Men**			
***income*** ***range*** ***(US dollars)***	***deaths***	***person-years***	***rate per 100 000 person-years***	***95% CI***	***deaths***	***person-years***	***rate per 100 000 person-years***	***95% CI***
0–4999	19	4704	404	(243, 630)	15	2567	584	(327, 964)
5000–9999	49	8603	570	(415, 763)	27	5495	491	(324, 717)
10000–14999	21	8912	236	(146, 361)	22	7539	292	(183, 441)
15000–19999	16	8932	179	(102, 290)	20	8281	242	(148, 372)
20000–24999	8	6305	127	(55, 250)	19	6113	311	(187, 485)
25000–29999	24	5016	478	(307, 713)	14	4622	303	(165, 509)
30000–34999	11	5854	188	(94, 336)	26	5836	445	(291, 655)
>= 35000	30	8561	350	(237, 501)	28	8698	322	(214, 467)

**Table 3:** Penalized spline Proportional Hazard model adjusted estimates of the association of income and mortality risk, healthy restricted population, age 18–64, NHANES mortality follow-up cohort, 1988–2001

	**Women (n = 6307)**			**Men (n = 5426)**		
**All-cause (women = 169, men = 181)**	***Chi-square***	***df***	***p-value***	***Chi-square***	***df***	***p-value***
Model 1. age, marital status, race/ethnicity	26	1.9	<0.001	10	2.1	<0.01
Model 2. model 1 + occupation and education	27	1.9	<0.001	11	2.1	<0.01
Model 3. model 2 + labor force participation	27	1.8	<0.001	11	2.1	<0.01
**heart disease (women = 30, men = 46)**						
Model 1. age, marital status, race/ethnicity	4.7	1.6	<0.1	27	1.7	<0.001
Model 2. model 1 + occupation and education	6.0	1.6	<0.05	13	1.6	<0.001
Model 3. model 2 + labor force participation	7.0	1.6	<0.05	13	1.6	<0.001
**Cancer (women = 63, men = 50)**						
Model 1. age, marital status, race/ethnicity	11	1.8	<0.01	10	1.4	<0.01
Model 2. model 1 + occupation and education	11	1.8	<0.01	8.4	1.5	<0.01
Model 3. model 2 + labor force participation	11	1.8	<0.01	8.7	1.4	<0.01
**Injury (women = 11, men = 32)**						
Model 1. age, marital status, race/ethnicity	10	1.0	<0.001	9.5	1.4	<0.01
Model 2. model 1 + occupation and education	8.2	1.0	<0.01	9.0	1.3	<0.01
Model 3. model 2 + labor force participation	7.3	1.0	<0.01	9.4	1.3	<0.01

**Table 4:** Proportional Hazard model fit statistics with alternative income terms, healthy restricted population, age 18–64, NHANES mortality follow-up cohort, 1988–2001

	**Women (n = 6307)**			**Men (n = 5426)**		
**All-cause (women = 169, men = 181)**	Likelihood ratio test	df	p-value	Likelihood ratio test	df	p-value
No income	26	12	0.01	67	12	1 × 10-9
Linear income	29	13	0.007	69	13	1 × 10-9
Log income	37	13	0.0005	70	13	9 × 10-10
Non-linear income	53	15	0.000003	80	15	8 × 10–11
**heart disease (women = 30, men = 46)**						
No income	8.6	12	0.7	27	11	0.004
Linear income	12	13	0.5	36	12	0.0003
Log income	10	13	0.7	30	12	0.003
Non-linear income	14	15	0.5	39	14	0.0003
**Cancer (women = 63, men = 50)**						
No income	20	11	0.05	36	11	0.0002
Linear income	21	12	0.05	36	12	0.0003
Log income	20	12	0.07	37	12	0.0003
Non-linear income	27	14	0.02	38	13	0.0003
**Injury (women = 11, men = 32)**						
No income	11	11	0.5	19	12	0.09
Linear income	11	12	0.5	19	13	0.1
Log income	11	12	0.5	20	13	0.09
Non-linear income	12	13	0.5	23	14	0.06

All models adjust for age (continuous), marital status (2 category), race/ethnicity (4 category), occupation (5 category) and education (as smoothed continuous term).

Note: Degrees of freedom between models for men and women may differ because when no events occurred within a particular strata, the covariate was omitted from all models within that gender and cause of death (this occurred for cancer mortality among women of the "other" race/ethnicity category, injury mortality for women of occupational category 3, heart disease mortality for men of the "other" race/ethnicity category, and cancer mortality for men of occupational category 5).

## Results

### Population

Table 1 shows the baseline (1988–1994) distribution of the full sample as compared to our restricted analytic sample of NHANES III participants without missing data who did not retire early or change jobs or go to part time work because of health related reasons. There were no substantial differences in the distribution of population characteristics between the full population and restricted samples, although there were slightly fewer individuals in the 45–64 age category in the restricted sample. Twenty-two percent had incomes below 50% of the median U.S. family size adjusted income (in this population, equivalent to $10,090), a level commonly used to define economic deprivation [28]. There were substantially fewer individuals not currently in the labor force in the restricted analytic sample.

### Income and all-cause mortality

Table 2 shows the unadjusted all-cause mortality rates (and 95% confidence intervals) by category of family equivalized income. Mortality rates are substantially higher among women and men in households with less than $10,000 a year of family income. Among women there is also an elevated rate of mortality in the $25,000-$29,999 income category, and among men in the $30,000-$34,999 category, but these elevated rates are not consistent across other income categories above $10,000 a year.

**Figure 1:**
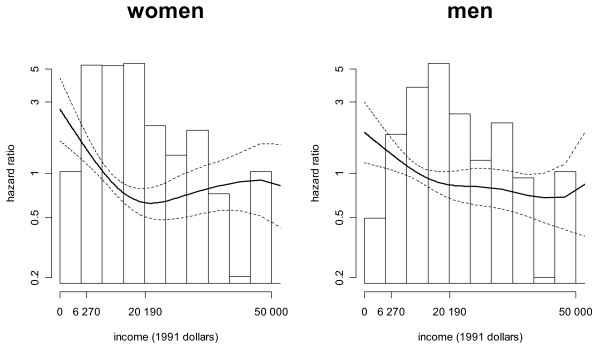
**Hazard ratios of all-cause mortality and income, ages 18–64, NHANES, 1988–2001**. Models control for age, race/ethnicity, marital status, occupational category and education (and income is adjusted for family size), and the population is restricted to individuals who did not die within one year of follow-up, retire early due to health reasons, change jobs due to health reasons, or change to part-time work due to health reasons. A hazard ratio of 1 is equivalent to the average risk of mortality across the income distribution. The overlaid histogram shows the population distribution by income level, and the labels of income level (in 1991 dollars) on the X-axis denote the family size equivalized US poverty line ($6,270) and the US equivalized median income ($20,190) as external standards of comparison. Dashed lines show 95% confidence intervals of the hazard ratio.

Figure 1 shows the smoothed hazard ratios (Y-axis) of all-cause mortality for women and men by level of equivalized family income (X-axis), for the population aged 18–64 at baseline. Models control for age, race/ethnicity, marital status, education and occupation, and the population is restricted to individuals who did not die within one year of follow-up, retire early due to health reasons, change jobs due to health reasons, or change to part-time work due to health reasons. The overlaid histogram shows the relative population distribution by income level, and the labels of income level on the X-axis denote the family size equivalized US poverty line ($6,270) and the US equivalized median income ($20,190) as external standards of comparison. Dashed lines show 95% confidence intervals of the hazard ratio. Because of the small number of events among the 8% of individuals with equivalized family income above $50,000, we have omitted from the plots the point estimate and confidence intervals above this level, even though we included these individuals in all analyses.

For all-cause mortality (Figure 1), among both women and men risk of mortality decreased with increasing income until near the median income level; and above this level, we observed no decreasing mortality hazard with income. Chi-square tests for the statistical significance of the non-linear associations between income and mortality hazard are given in table 3. The Chi-square test statistic, degrees of freedom (df) and p-value are shown for the associations in figure 1 (Model 2) as well as for models controlling only for age, marital status and race/ethnicity (Model 1), and for models additionally controlling for labor force participation (Model 3). In all models (except model 1 for heart disease among women) the Chi-square test for a non-linear association of income with mortality was significant at the alpha = 0.05 level (Table 3).

### Income and cause-specific mortality

**Figure 2:**
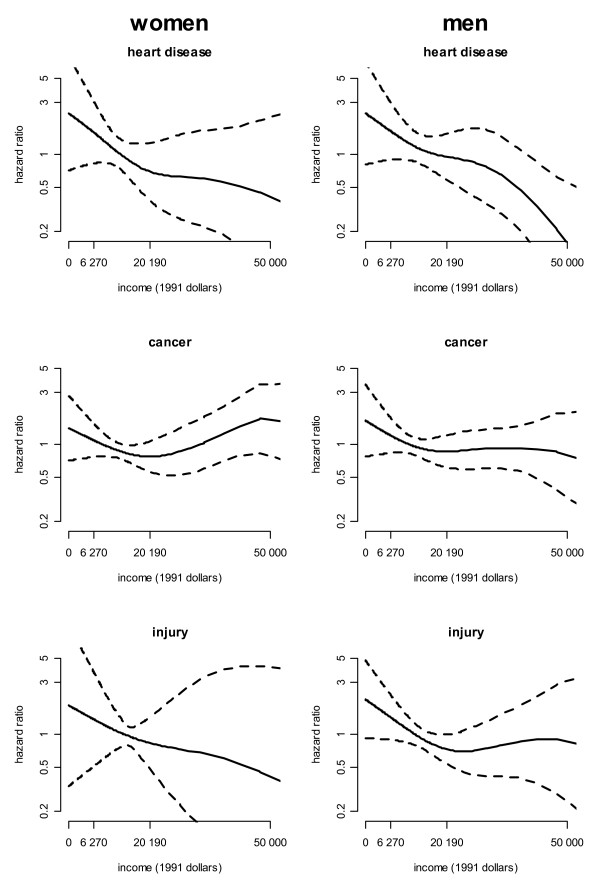
**Hazard ratios for cause-specific mortality and income, ages 18–64, NHANES, 1988–2001**. Models control for age and race/ethnicity, marital status, occupational category and education (and income is adjusted for family size), and the population is restricted to individuals who did not die within one year of follow-up, retire early due to health reasons, change jobs due to health reasons, or change to part-time work due to health reasons. A hazard ratio of 1 is equivalent to the average risk of mortality across the income distribution. The labels of income level (in 1991 dollars) on the X-axis denote the family size equivalized US poverty line ($6,270) and the US equivalized median income ($20,190) as external standards of comparison. Dashed lines show 95% confidence intervals of the hazard ratio.

For cause-specific mortality (Figure 2), risk of death due to heart disease among women showed a similar pattern as was observed for all-cause mortality, where the income-mortality association was strongest below median income. For men, the risk of mortality decreased in a generally linear pattern. For death due to cancer, women and men exhibited different non-linear risk with income. Among persons below median family income, risk of mortality due to cancer declined slightly with higher levels of income for women and men. Among persons above median family income, risk was not associated with income among the men, but increased among women. Lastly, for death due to injury, risk decreased most strongly below median income, with some further decrease in risk above this point among women but not among men.

### Alternative models of income and mortality

We repeated the analyses shown in Figures 1 and 2 both not controlling for occupational category and years of education (Model 1) and additionally controlling for labor force participation (Model 3) and obtained similar results (Table 3). Among women and men, all-cause mortality remained significantly associated with income in a non-linear manner for all outcomes in all three models.

We also examined the extent to which specifying a non-linear functional form of income improved the overall model fit for prediction of mortality as compared to: 1) baseline covariates and no income variable; 2) baseline covariates and a linear income variable; or 3) baseline covariates and a log transformation of income. We did so by comparing the likelihood ratios of each model, taking into account increased degrees of freedom of income and the non-linear income models (Table 4). A lower p-value represents a stronger rejection of the null hypothesis of no difference between a null model with no predictors and the full models. For all-cause mortality (among women and men) and injury (among men) the models with non-linear income are the best fit to the data. For heart disease (among men and women), cancer (among men) and injury (among women) a non-linear income model is an equally good fit to the data as a linear income model (heart disease), a linear or log income term (cancer among men), or any of the other models for injury among women.

## Discussion

Among US women and men age 18 to 64 at baseline, with follow-up of up to 13 years, we found evidence of a generally stronger associations of income with all-cause mortality at the lower end of the income distribution, i.e., under median income. Similar patterns occurred for deaths due to cancer and injury; by contrast, a more linear association across the full income range was evident for death due to heart disease. These results are unlikely to be substantially driven by contemporaneous effects of illness on income because of the restrictions of our sample to individuals with more than one year of follow up who had not ever changed jobs, changed to part-time work, or retired early due to health reasons. In fact, our results are likely a conservative estimate of the association due to the potential effects of income on illness, given that we restricted our analysis to a healthy sample that has not left the labor force due to health reasons.

Our results for all-cause mortality are generally consistent with the previously reported logarithmic functional form of association with income [9,10]. However, our results expand on this observation in three important ways. First, we find that the shape of association varies by type of mortality. Second, based on the overall fit of models with either a log function of income or a non-linear function of income, the appropriateness of a log function for modelling income and mortality holds most closely for only cancer mortality among men and injury mortality among women. Third, based on visual inspection of the plotted income-mortality hazard ratios we identify median income level as a critical point of inflection for outcomes where substantial non-linear associations were found.

These results should be considered within the context of several study limitations. First, the data we used lack specific income categories above $50,000 a year, which limits our understanding of the impacts of income for the 8% of families that had the highest equivalized income. Our estimates at the upper end of the income distribution are less precise, as indicated by the widening confidence intervals in the plots, and the point estimates in these regions should be interpreted cautiously. A second limitation of this analysis is that income is measured at only one point in time, thus not capturing household income dynamics that influence health outcomes [29-31]. This also results in a potential mismatch between income and social class related exposures and the relevant time periods for disease etiology. However, as income level of households are correlated across time, a recent measure of income does capture an important aspect of households' socioeconomic position [30], and one that is strongly correlated with permanent income. A third limitation is that as with any non-experimental study the associations observed may be due to residual confounding rather than a causal relationship between income and mortality. For example, we lack information on wealth and debt, additional markers of socioeconomic position that have been demonstrated to affect health [28,32]. This may result in some residual confounding by social class that may contribute to bias in our estimates of the associations between income level and mortality risk [33].

Based on a qualitative inspection of the smoothed plots of the income-mortality association, and the overall tests of model fit, we have shown that a non-linear association with a stronger association below median income is the most prevalent pattern of association. There were however variations in this association by cause and by gender. For heart disease, in particular among men, there appears to be less of a threshold at median income. While this may be due to mortality risk for heart disease more evenly distributed across the income distribution, an alternative explanation is that we have limited power to detect the shape of the association due to a relatively small number of heart disease events above median income (as confidence intervals indicate). Supporting this later speculation is prior work examining a two slope model of income and cardiovascular mortality that has shown there is a stronger association at lower income levels [10].

While significant associations were observed for both men and women, and the shape of the relation was similar for all-cause, heart disease and injury mortality, there were different associations observed among women and men for cancer above median income – no association for men, and a slightly increasing association among women. Tests of overall model fit showed that while a linear or logarithmic model of income was an equally good fit to the data among men, a non-linear model was a better fit among women. This difference may be due to the positive association between socioeconomic position and rates of breast cancer mortality in women that does not exist as strongly for any site of cancer in men [34,35]. These differing results by cause and gender are consistent with the context-, time-, and cause-contingent nature of social gradients; empirical findings indicate that income-mortality associations are not fixed, but rather vary across cultures and centuries [36].

Our results are generally consistent with the two other US studies that examined the shape of the income-mortality association [9,10], despite the fact that these studies were not able to eliminate individuals who had health problems that may have effected their income. While our results not adjusting for labor force partipation are similar to those observed by Blakely et al. in New Zealand, this study finds that after adjusting for labor force participation the association of mortality with income is markedly attenuated, in contrast with our findings [8]. This may be due to our restricted healthy sample, our non-linear method of analysis, or due to stronger effects of labor force participation on mortality in New Zealand as compared to the United States. Our finding of a non-linear shape of association contrast with findings from Finland, where Martikainen et al report a linear shape of association between income and mortality [6]. While this may be due to differing access to benefits and social services in the U.S. and Finland, Blakely et al. note that if an absolute level of income was used to assess the shape of the association, the results of Martikainen et al are consistent with a flattening of risk at upper income levels[8]

The results presented have implications for understanding the etiological links between income and mortality. Based on the observed associations, income disparities in mortality chiefly among lower income populations (below the median income) appear to be driving the commonly reported socioeconomic gradients in all-cause, cancer among men and injury mortality. These findings also underscore why efforts to address income disparities in mortality cannot be restricted simply to persons below the US poverty line but instead should include persons with income at least up to the median level. The difference in size of these two populations is large: in 1991, the mid-point of the income data collection, 13% of families in the U.S. were below the poverty line (equal to $10,860 for a three person family) as compared to 50% of families below the median family income (equal to $35,940), an absolute difference of 37% of US families, and similar to what we have in our study population (Table 1). While anti-poverty policies and programs that include individuals up to either 125%, 150% or 185% of poverty level are common (185% of the poverty level is equivalent to $20,090 for a family of three), the additional population between the 185% poverty level and median income, equivalent to approximately 22% of US families, constitute a large segment of the population that potentially is not benefiting from efforts to reduce health and economic disparities. Suggesting this could be done, benefits from the earned income credit, which specifically address income disparities, reach beyond 185% of the poverty level [37].

## Conclusion

In the US context, in adults aged 18–64 at baseline, the non-linear risk of mortality with income arises from the stronger relationship between income level and mortality among lower compared to higher income populations. This evidence is supportive of the hypothesis that policies to improve the health of individuals among the lower half of the income distribution will have the most impact on reducing US income-based disparities in mortality, although from our data we cannot establish why this association exists. Second, if the associations presented are not due to residual confounding, measurement error, or unaccounted for reverse causation, they may also have implications for the importance of income in contributing to premature mortality. Future studies determining the pathways connecting income and mortality will benefit from a consideration of where in the income distribution the burden of disparity exists, and that this association varies by cause.

## Competing interests

The authors declare that they have no competing interests.

## Authors' contributions

DHR designed the study, conducted the analysis, and lead the writing of all sections of the manuscript. NK contributed to the design of the study and contributed to writing all sections of the manuscript. BC contributed to the design of the study, data analysis and contributed to writing all sections of the manuscript. LFB contributed to the design of the study and contributed to writing all sections of the manuscript.

## Pre-publication history

The pre-publication history for this paper can be accessed here:



## Supplementary Material

Additional File 1 **Table of occupational classifications.** The data provided describes the categorization of the NHANES III occupational categories in order to create the occupation variable used as a covariate in the analysis. Click here for file
