# Supplementary material for: The non-linear risk of mortality by income level in a healthy population: US National Health and Nutrition Examination Survey mortality follow-up cohort, 1988–2001
Source: BMC Public Health. 2008 Nov 10;8:383. doi: 10.1186/1471-2458-8-383 (PMC2587469; doi:10.1186/1471-2458-8-383)
Supplement: Additional File 1 — Table of occupational classifications. The data provided describes the categorization of the NHANES III occupational categories in order to create the occupation variable used as a covariate in the analysis. [file 1471-2458-8-383-S1.doc]

# Additional file

### Table of occupational classifications

| **Category** | | **NHANES III occupational category item description and code** |
| --- | --- | --- |
| White collar and  professional | | Executive, administrators, and managers (1), management related occupations (2), engineers and scientists (3), health diagnosing, assessment and treating occupations (4), teachers (5), writers, artists, entertainers, and athletes (6), other professional specialty (7), supervisors and proprietors, sales occupations (9), farm operators, managers, and supervisors (25) |
| White collar, semi-routine | | Technicians and related support occupations (8), sales representatives, finance, business, and, commodities except retail (10), secretaries, stenographers, and typists (12), information clerks (13), records processing occupations (14), material recording, scheduling, and distributing clerks (15), miscellaneous administrative support occupations (16), health service occupations (22) |
| Blue collar,  high skill | | Vehicle and mobile equipment mechanics and repairers (28), other mechanics and repairers (29), construction trades (30), extractive and precision production occupations (31), armed forces (NHANES III variable HAS17=2) |
| Blue collar, semi-routine | Sales workers, retail and personal services (11), private household occupations (17), protective service occupations (18), waiters and waitresses (19), cooks (20), miscellaneous food preparation and service occupations (21), cleaning and building service occupations (23), personal service occupations (24), farm and nursery workers (26), related agricultural, forestry, and fishing occupations (27), textile, apparel, and furnishing machine operators (32), machine operators, assorted materials (33), fabricators, assemblers, inspectors, and samplers (34), motor vehicle operators (35), other transportation and material moving occupations (36), construction laborers (37), laborers, except construction (38), freight, stock, and material movers, hand (39), other handlers, equipment cleaners, and handlers (40) | |
| Never worked | Never worked (NHANES III variable HAS17=1) | |
